# Computational analysis of missense variant CYP4F2*3 (V433M) in association with human CYP4F2 dysfunction: a functional and structural impact

**DOI:** 10.1186/s12860-023-00479-0

**Published:** 2023-05-09

**Authors:** Mahvash Farajzadeh-Dehkordi, Ladan Mafakher, Fatemeh Samiee-Rad, Babak Rahmani

**Affiliations:** 1 Department of Molecular Medicine, Faculty of Medical School, Qazvin University of Medical Sciences, Qazvin, Iran; 2 Thalassemia & Hemoglobinopathy Research center, Health research institute, Ahvaz Jundishapur University of Medical Sciences, Ahvaz, Iran; 3 Department of Pathology, Faculty of Medical School, Qazvin University of Medical Sciences, Qazvin, Iran

**Keywords:** Damaging variant, CYP4F2*3, V433M variation, Warfarin, Vitamin K1, In silico, Molecular modeling, Molecular dynamics simulations

## Abstract

**Background:**

Cytochrome P450 4F2 (CYP4F2) enzyme is a member of the CYP4 family responsible for the metabolism of fatty acids, therapeutic drugs, and signaling molecules such as arachidonic acid, tocopherols, and vitamin K. Several reports have demonstrated that the missense variant CYP4F2*3 (V433M) causes decreased activity of CYP4F2 and inter-individual variations in warfarin dose in different ethnic groups. However, the molecular pathogenicity mechanism of missense V433M in CYP4F2 at the atomic level has not yet been completely elucidated.

**Methods and results:**

In the current study, we evaluated the effect of the V433M substitution on CYP4F2 using 14 different bioinformatics tools. Further molecular dynamics (MD) simulations were performed to assess the impact of the V433M mutation on the CYP4F2 protein structure, stability, and dynamics. In addition, molecular docking was used to illustrate the effect of V433M on its interaction with vitamin K1. Based on our results, the CYP4F2*3 variant was a damaging amino acid substitution with a destabilizing nature. The simulation results showed that missense V433M affects the dynamics and stability of CYP4F2 by reducing its compactness and stability, which means that it tends to change the overall structural conformation and flexibility of CYP4F2. The docking results showed that the CYP4F2*3 variant decreased the binding affinity between vitamin K1 and CYP4F2, which reduced the activity of CYP4F2*3 compared to native CYP4F2.

**Conclusions:**

This study determined the molecular pathogenicity mechanism of the CYP4F2*3 variant on the human CYP4F2 protein and provided new information for understanding the structure-function relationship of CYP4F2 and other CYP4 enzymes. These findings will aid in the development of effective drugs and treatment options.

## Introduction

Since its clinical approval, warfarin remains the mainstay of an oral vitamin K antagonist agent with verified efficacy for preventing and treating patients with thromboembolic diseases [1]. The clinical utility of warfarin is growing worldwide because of the increased frequency of cardiovascular disorders and the accelerated aging of the population. However, the considerable interindividual variation and narrow therapeutic index of warfarin dosing make warfarin treatment more difficult [2]. The variability in response to warfarin dose is associated with environmental, demographic, clinical, and mainly genetic factors [3, 4].

Currently, genetic factors, especially single nucleotide polymorphisms (SNPs) in cytochrome P450 2C9 (CYP2C9) and vitamin K epoxide reductase complex 1 (VKORC1) genes are related to the both pharmacokinetics and pharmacodynamics of warfarin which could account for 15% and 25% of warfarin dose variation, respectively [5–7]. Furthermore, the cytochrome P450 4F2 (CYP4F2) functional missense variant, CYP4F2*3 (c.1297G > A, p. V433M, rs2108622) was found to moderately contribute to the variability in warfarin doses in various ethnic groups [8]. Individuals carrying the homozygote (TT) and heterozygote (CT) forms of CYP4F2*3 alleles require a higher dose of warfarin to get the same targeted anticoagulant effect as individuals with the wild-type genotype (CC) [9, 10].

The gene of CYP4F2 located on chromosome 19 p13.12 encodes the CYP4F2 enzyme. This enzyme is expressed in the kidneys, liver, and human enteric microsomes [11, 12] and is involved in ω- hydroxylation of long-chain fatty acids, leukotriene-B4, arachidonic acid, tocopherols, and vitamin K [13–16]. In addition, CYP4F2 metabolizes the ester prodrug gemcitabine and the antiparasitic pafuramidine [17, 18]. Moreover, CYP4F2 has an essential biological function in activating signaling compounds and regulating inflammation. In addition, the increased levels of CYP4F2 protein are associated with different types of carcinomas [19]. This enzyme catalyzes the hydroxylation of vitamin K1 (VK1) to its hydroxylated form via vitamin K oxidase activity. This process, characterized as the “siphoning” pathway, occurs when vitamin K1 is in excess [14]. The amount of active VK1 is essential not only for the stimulation of clotting factors and maintenance of warfarin dosing but also for many physiological processes affected by the CYP4F2 genotype [20]. Several experimental studies have attempted to predict the molecular mechanism of the CYP4F2*3 variant that influence the metabolic activity of the CYP4F2 enzyme [14, 15, 21, 22]. McDonald et al.(2009) reported that the CYP4F2*3 variant induces a reduction in enzyme activity by affecting either an increased rate of protein degradation or a decreased rate of CYP4F2 translation [14]. Zhang et al.(2017) discovered a significant relationship between the CYP4F2*3 variant and an increase in mRNA expression of CYP4F2, describing more than 12% of the variance in CYP4F2 mRNA expression [22]. However, the details of molecular mechanisms of the CYP4F2*3 variant that may affect protein structure and function are still unknown.

Compared with laboratory-based experiments for evaluating the effects of SNPs, computational approaches provide an appropriate platform for assessing genetic mutations for their pathogenic effects and determining their underlying molecular mechanisms [23–25]. In addition, understanding the three-dimensional structure of a protein is important to understand the mechanism by which a protein performs its function. Over the past few years, the molecular dynamics (MD) simulations method has become a valuable tool for comprehending the impact of mutations on the operation and conformation of various proteins at an atomistic level [26, 27].

Therefore, in the current study, we aimed to identify the effect of the CYP4F2*3 variant on the function, stability, and structure of CYP4F2 at the atomic level using bioinformatics prediction tools, MD simulations, and molecular docking calculations. Overall, the current research provides crucial information on atomic-level alterations, which are otherwise difficult to evaluate using experimental approaches, and these results might function as a bridge to connect the in silico and clinician’s resources in designing effective drugs and individual treatment options.

## Materials and methods

### Data collection

The FASTA sequence of the human CYP4F2 protein was obtained from UniProt (ID: P78329) [28]. The clinical information of variant CYP4F2*3 and its relation to warfarin dose was retrieved from the PharmGKB (ID: PA166169424) [29] and PubMed databases [8–10].

### Structural modeling and verification

Because the three-dimensional structure of human CYP4F2 does not exist, homology modeling was performed using the I-TASSER web server for both native and mutant form [30]. Then, the quality of the modeled structure was evaluated using Procheck, ERRAT, and Verify3D in the SAVES web server (https://saves.mbi.ucla.edu/). The Procheck platform checks the stereochemical quality of the model using a Ramachandran plot [31]. A protein structure with more than 80% amino acids in the core and allowed regions indicated high quality. The ERRAT webserver statistically analyzed the non-bonded interactions between different atom types in the protein structure [32]. Verify3D webserver has characterized the compatibility of an atomic model (3D) with its amino acid sequence (1D) by analyzing a structural class according to its location and environment (alpha, beta, loop, polar, nonpolar, etc.) [33]. A protein structure with a Verify3D value greater than 80% is defined as having good compatibility with 1D and 3D protein structures and good quality. ProsA is a web server program that is frequently used to check 3D models of protein structures for possible errors. The overall quality score computed by ProSA for a particular protein structure is represented in a plot that indicates the scores (Z-score) of all experimentally characterized protein chains recently available in the Protein Data Bank (PDB) [34]. The protein structure Z-score, which is located in the experimentally characterized protein structure range, indicates a high-quality protein structure.

### Protein stability prediction

Five different prediction tools, I-mutant2, MUpro, DUET, SDM, and mCSM were utilized to identify the structural impact of the CYP4F2*3 (V433M) variant on the CYP4F2 protein. The server of I-Mutant 2.0 (http://folding.biofold.org/i-mutant/i-mutant2.0.html) estimates the impact of amino acid substitution on protein stability via a support vector machine-based method. This program was trained on ProTherm, the most comprehensive dataset of experimental information on protein mutations [35]. The MUpro tool (https://mupro.proteomics.ics.uci.edu/) uses neural networks and support vector machine methods to assess any alteration in the stability of the protein with approximately 84% accuracy [36]. Three structure-based algorithms, DUET, SDM, and mCSM (http://biosig.unimelb.edu.au/duet/) are closely intertwined algorithms and their input can be supplied together in the form of a native protein structure, while separately bringing up the position of relevant SNP [37].

### Protein property and evolutionary conservation analysis

GETAREA is a structure-based tool (https://curie.utmb.edu/getarea.html) used to determine the effect of the V433M variant on the solvent accessibility surface area (SASA) of the CYP4F2 protein. The percentage of side-chain surface area to random coil value per residue (% SASA) estimates whether the residue is buried in the solvent or exposed [38]. The server of Project HOPE (http://www.cmbi.ru.nl/hope/input/) by utilizing the integrating information from the UniProt database and Distributed Annotation System (DAS) servers, predicts the effect of amino acid substitution on the protein structure [39]. To visualize the impact of the V433M variant at the structural level, Chimera 1.13 software was used. This software is an extended system for the interactive visualization and evaluation of molecular structures and related data [40]. The ConSurf web server (http://consurf.tau.ac.il) was used to define the conservation sites in the protein compared to its homologs during evolution through conservation scores ranging from 1 to 9, which are classified as variable, intermediate, and conserved [41].

### Functional impact prediction

To determine the functional consequences of the V433M variant on the CYP4F2 protein, five different tools including PolyPhen-2, FATHMM-MKl, PANTHER-PSEP, CADD, and PhD-SNP^g^ were employed. The server of PolyPhen-2 (http://genetics.bwh.harvard.edu/pph2/) determines the possible effect of amino acid substitution on protein properties, based on position-specific independent count (PSIC) scores [42]. The difference between the scores categorizes the variants as probably damaging, possibly damaging, or benign. FATHMM-MKL (https://fathmm.biocompute.org.uk/) is a web-based tool in which a prediction score (p-value) above 0.5 identifies the variant that has a harmful effect on protein function [43]. Based on the substitution position-specific evolutionary conservation (subPSEC) score, PANTHER (http://pantherdb.org/) predicts the probability of a variant generating a harmful effect on the protein. If the score of subPSEC is ≥ 0.5, the variant is deemed deleterious [44]. CADD (http://cadd.gs.washington.edu/) as a linear kernel support vector machine-based, predicts functionally important variants using a Phred-scale C score. CADD considers amino acid substitutions as the most deleterious when they have a C-score of 20 or greater (≥ C20) [45]. PhD-SNP^g^ (http://snps.biofold.org/phd-snpg./) is a machine learning-based server that identifies the impact of variants in both non-coding and coding regions using evolutionary data [46].

### Molecular dynamic (MD) simulation analysis

Gromacs 2019 was applied to run an MD simulation with an optimized potential for liquid simulations (OPLS) force field. Simple point charge (SPC) water was added to the simulation box cubic with a 10 A° [47, 48]. The simulation systems’ charges were neutralized using Na^+^ and Cl^−^ ions. To minimize the simulation system, a steepest descent minimization integrator with a maximum force of less than 100 KJ.mol^− 1^. nm^− 1^ and 5000 minimization steps were used. The simulation system was then equilibrated by a constant number of particles, volume, and temperature (NVT) and a constant number of particles, pressure, and temperature (NPT) ensemble. For NVT equilibration through 100 picoseconds, the leap-frog integrator and linear constraint solver (LINCS) algorithm were used [49]. The PME (particle mesh Ewald) algorithm with a grid spacing of 0.16 nm and a radius cutoff of 1.0 nm was considered for calculating electrostatic interaction. The equilibrated system was put into an MD simulation with a time step of 2 femtoseconds (fs) for 200 nanoseconds (ns) of simulation. The output trajectories were analyzed using root-mean-square deviation (RMSD), radius of gyration (Rg), solvent access surface area (SASA), total number of intramolecular hydrogen bonds, root-mean-square fluctuation (RMSF), secondary structure by DSSP, principal component analysis (PCA) and free energy landscapes (FELs) to determine the stability and protein structure changes during simulation.

### Molecular docking analysis

As VK1 is a substrate for the CYP4F2 protein and its effective interaction is essential for the proper function of CYP4F2, molecular docking calculations were performed using the HADDOCK web server to determine the effect of the CYP4F2*3 variant on the native protein structure [50]. This web server uses a flexible docking approach to define the best protein-ligand interaction mode. In addition, the PRODIGY web server was applied to compute the binding affinity of the protein-small molecule [51] and the bond-type analyses were done by Discovery Studio 4.5 [52].

## Results

### Structural modeling validation and quality estimation

**Fig. 1:**
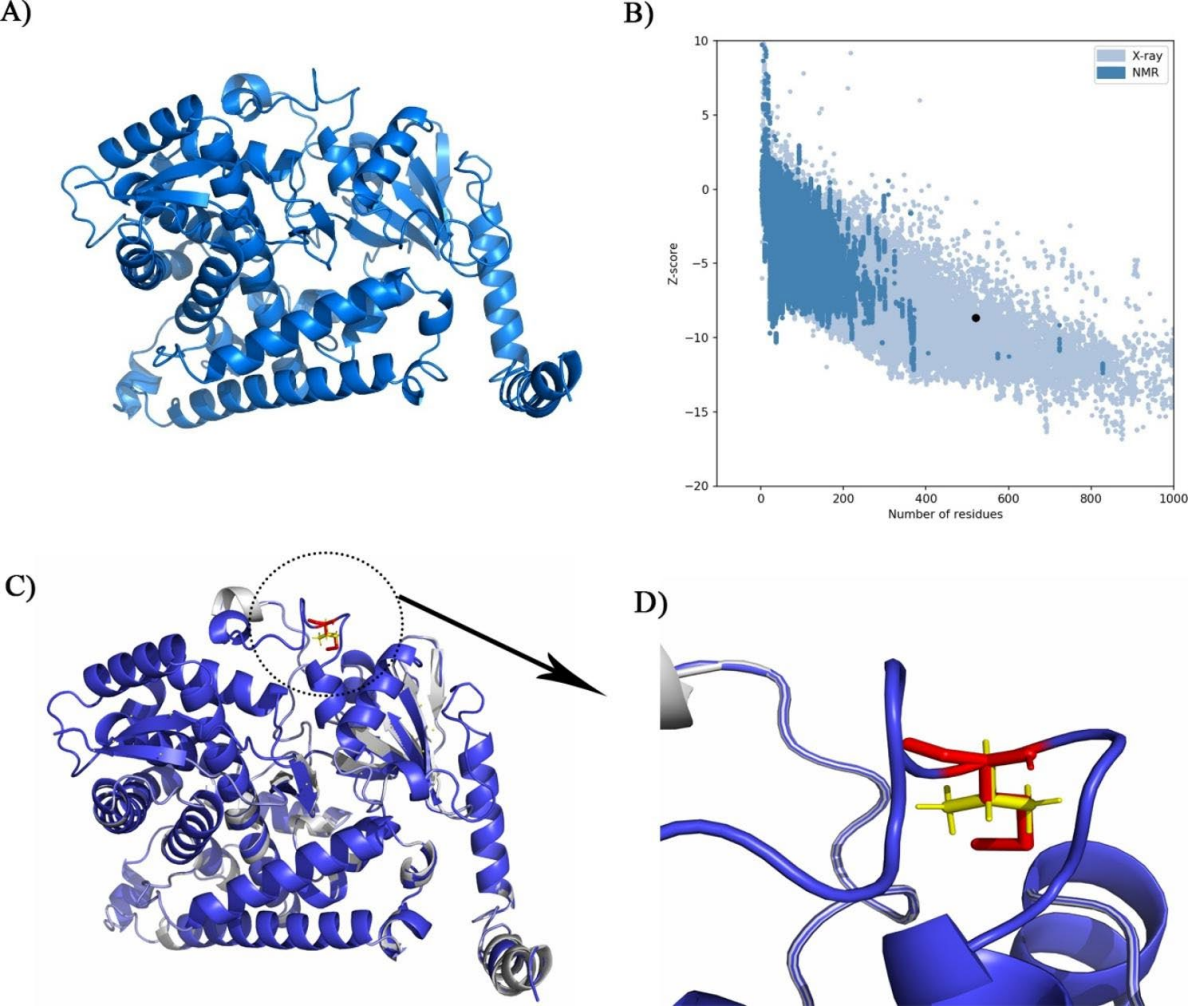
** A**) Tertiary structure of modeled CYP4F2 protein by I-TASSER web server in cartoon view. **B**) ProSA plot of quality assessment of CYP4F2 structure compared to proteins characterized experimentally. Data showed that CYP4F2 (shown in a black dot in the plot) is located in the region of the protein structure identified in the lab. **C**)Tertiary structure alignment of V433M (blue) and native (gray) form. A red surface color characterizes the position of the V433M variant. **D**) Close up view of structural alignment of native and mutant form. Data shows that methionine (red stick) has larger more buried side chain than valine (yellow stick)

Owing to the lack of tertiary structure of CYP4F2, homology modeling was applied using the I-TASSER webserver (Fig. 1A). The quality of the predicted structure was analyzed using different web servers (Table 1). Checking the quality of a model by Ramachandran plot with Procheck web server illustrated that more than 80% of the protein structures were located in the core and allowed regions, indicating the high physicochemical quality of the protein structure. A Verify3D value of more than 80% showed high compatibility between the primary and tertiary structures of CYP4F2. The ERRAT rate of 91.01 is interpreted good overall quality of protein structure. The Z-score of -8.66 was located in regions of proteins that were identified experimentally, indicating the high quality of the predicted model of CYP4F2 (Fig. 1B). The position of wild-type residue (V433) and mutant-type residue (M433) of V433M variant in CYP4F2 protein was shown in Fig. 1C and D.

**Table 1:** Physicochemical quality assessment of CYP4F2 protein structure

Model	Procheck				Verify3D	ERRAT	ProsA Z-score
	**Core**	**Allowed**	**Generally allowed**	**Disallowed**			
CYP4F2	76.9%	18.1%	2.6%	2.4%	87.12%	91.01	-8.66

### Protein stability prediction

I-mutant2, MUpro, DUET, SDM and mCSM tools predict protein stability changes in the CYP4F2*3 (V433M) variant based on the ΔΔG value (change in Gibbs free energy). The ΔΔG value is a metric used to calculate the impact of a single nucleotide variant on protein stability. A ΔΔG score less than zero indicated lower stability. From the results illustrated in Table 2, the CYP4F2*3 (V433M) variant exhibited reduced protein stability. Destabilization of a protein structure can change its biological function and disrupt the signaling cascades and normal pathways of the protein [53].

**Table 2:** Stability prediction of V433M variant on CYP4F2 protein by using five prediction tools

dbSNP ID	Amino acid change	I-mutant2	MUpro	DUET	mCSM	SDM
		ΔΔG (kcal/mol)	ΔΔG (kcal/mol)	ΔΔG (kcal/mol)	ΔΔG (kcal/mol)	ΔΔG (kcal/mol)
CYP4F2*3 (rs2108622)	V433M	-2.18	-1.129	-0.605	-0.391	-1.1

### Protein property and evolutionary conservation analysis

GETAREA calculates the SASA of individual residues. The threshold is described as %SASA > 50 and %SASA < 20, indicating exposed and buried residues to the solvent, respectively [38]. Based on the results obtained from the GETAREA online for the V433M variant, both wild-type (V433) and mutant-type (M433) amino acids were buried or located in the interior of the CYP4F2 protein. However, the mutated residue had a lower % SASA value (% 5.9) than the wild-type V433 (% 9). This result was compatible with the deep view of the locations of the wild-type (V433) and mutant-type (M433) amino acids (Fig. 1D), which showed that M433 residue had more buried side chain than V433 residue.

The Project HOPE server investigated the effect of the V433M variant on the physiochemical characteristics, intermolecular interactions, and functional and structural properties. It was predicted that the wild-type residue (V433) would be replaced by a mutant-type residue (M433), which has a larger side chain than the V433 moiety and may not fit into the new position. For physical and structural reasons, if a substituted amino acid does not fit into a protein, it leads to structural changes that are harmful [54]. Furthermore, the Project HOPE server predicted that the V433 residue is located near a highly conserved region and in contact with residues in a domain that is important for protein activity.

**Fig. 2:**
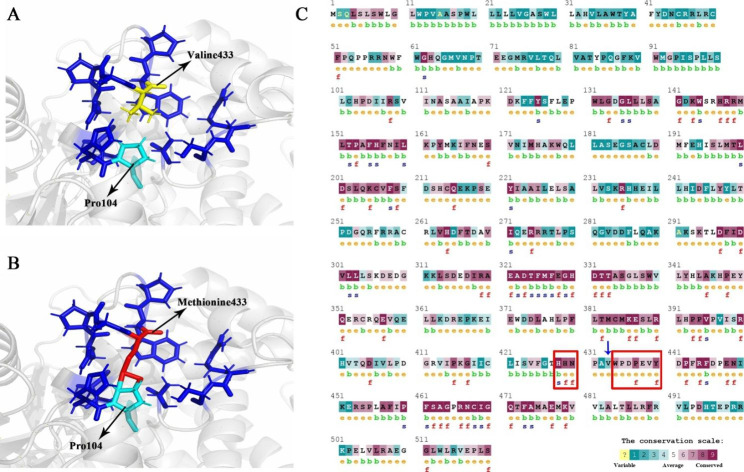
Structural and conservation analysis of CYP4F2 in native and mutant form. **A**) Structure analysis of native form of CYP4F2 with its neighboring residues. **B**) Structure analysis of CYP4F2*3 (V433M) variant with its neighboring residues. **C**) The conservation analysis of CYP4F2 illustrates that V433 residue (characterized by blue arrow) is located near conserve region of CYP4F2 protein which shows with red rectangular

Structural analysis of neighboring amino acids around the V433 residue in less than 5-angstrom regions using the Chimera software revealed that amino acids including H103, P104, T427, N430, P431, A432, W434, P435, R453, S454 and A457 were located close to the V433 residue. For the V-to-M conversion at position 433, the mutated residue with a larger side chain than the wild-type residue caused a clash score with neighboring residues, especially with the P104 residue when compared to wild-type (Fig. 2A and B).

The ConSurf web server determined that V433 residue is positioned in the variable site (conservation score of 2) in the protein, while it is located close to residues that are highly conserved in protein when compared to homologous proteins of CYP4F2 during evolution (Fig. 2C).

### Functional impact prediction

Five tools have been utilized to identify the functional effect of the CYP4F2*3 (V433M) variant on the CYP4F2 protein (Table 3). PolyPhen-2, FATHMM-MKl, PANTHER-PSEP, CADD, and PhD-SNP^g^ tools have identified the CYP4F2*3 (V433M) as a deleterious variant.

**Table 3:** Pathogenicity evaluation of V433M variant on CYP4F2 protein by using bioinformatics tools

dbSNP ID	Amino acid change	PolyPhen-2		FATHMM-MKl		PANTHER-PSEP		CADD		PhD-SNP^g^	
		Prediction	Score	Prediction	Score	Prediction	Score	Prediction	Score	Prediction	Score
CYP4F2*3 (rs2108622)	V433M	Possibly damaging	1.0	AEFBHCI	0.754	Probably damaging	0.74	Deleterious	22.3	Pathogenic	0.865

### MD simulation findings

**Fig. 3:**
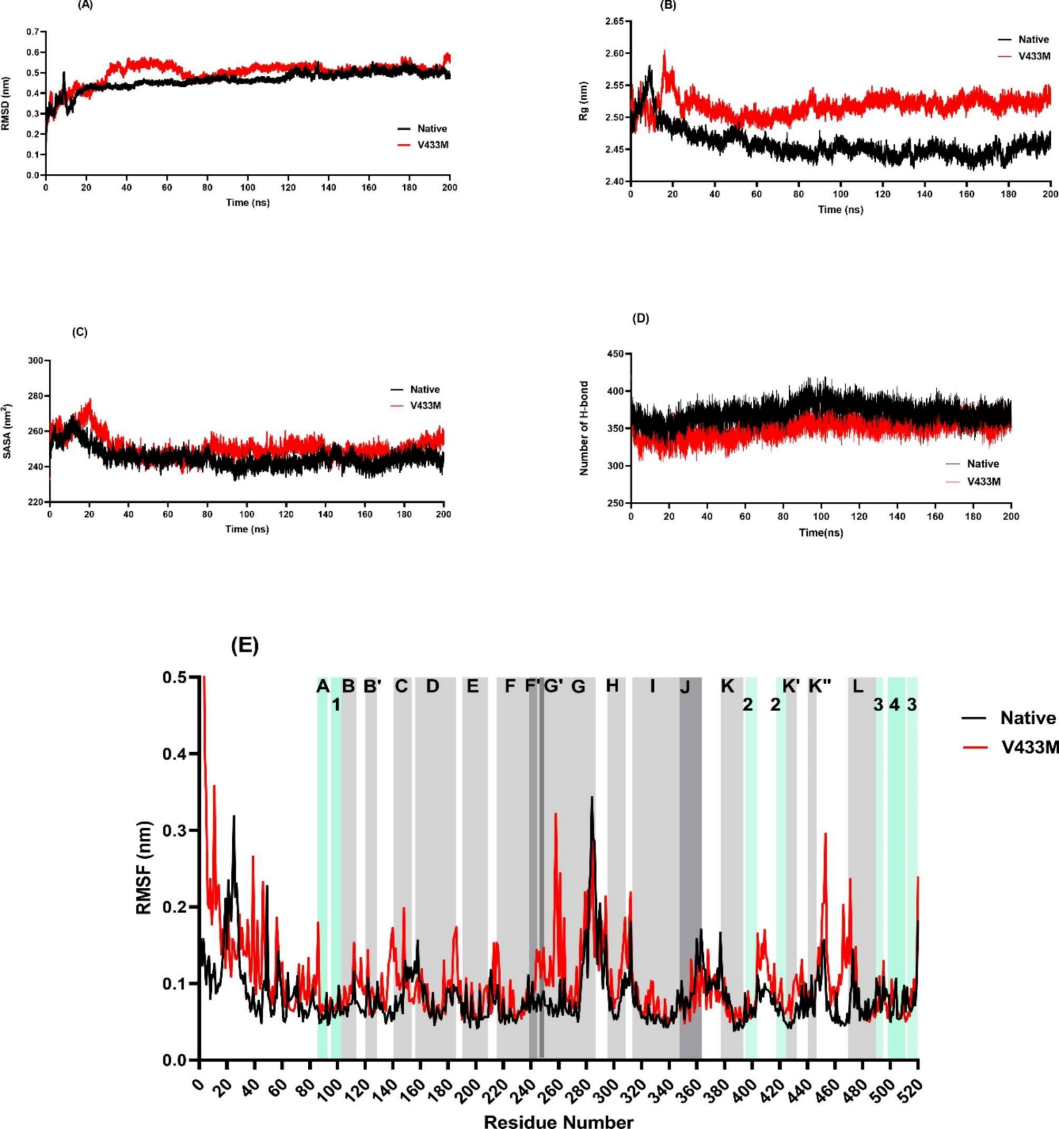
Result of structural dynamics of native CYP4F2 (black color) and variant V433M (red color) as a function of time obtained by 200 ns of the MD simulations. **A**) RMSD values, **B**) Rg values, **C**) SASA calculations, **D**) total number of H-bond count and **E**) Residual fluctuations of the Cα atoms as a function of residue number. The native form of CYP4F2 is shown in black and the V433M variant is in red. The gray and blue blocks show α-helices (indicate with letters) and β-strands (identify with numbers), respectively

200 ns MD simulations were conducted to analyze the deviation between native and CYP4F2*3 (V433M) variant proteins in physiological environments (Fig. 3). RMSD deviations produced during MD simulations were used to estimate the stability of the protein [55]. As presented in the RMSD plot (Fig. 3A), the native protein attains a relatively stable conformation after 20 ns. In comparison, the V433M variant was stable after approximately 70 ns of simulations suggesting that the V433M variant experienced more significant structural rearrangement before reaching a stable structure. In addition, the RMSD average values verified that the mutant structure had a slightly higher RMSD value (0.49 ± 0.05 nm) than the native structure (0.46 ± 0.04 nm), which means that the mutant form caused instability in the protein structure of CYP4F2.

Rg is an essential parameter for evaluating the dynamic adaptability of proteins and other biopolymers [56]. The Rg plot showed that the V433M mutant fluctuated more than that of the native form (Fig. 3B). In addition, the average Rg value analysis showed that the V433M mutation had a higher average Rg value (2.45 ± 0.02 nm) than the native protein (2.51 ± 0.01 nm) during the simulation time. This result indicates that the V433M mutation is less compact and more flexible than native CYP4F2.

SASA denotes the portion of the macromolecule’s surface accessible to the water solvent [57]. As shown in Fig. 3C, SASA analysis revealed that the V433M mutation had a higher average SASA value (250.74 ± 6.13 nm) than native CYP4F2 (245.17 ± 5.48 nm), suggesting that structural rearrangements in the V433M mutation resulted in increased protein expansion.

The total number of intramolecular hydrogen bonds was estimated during the 200 ns MD simulation as shown in Fig. 3D. From the analysis, it can be observed that native CYP4F2 forms a greater number of H-bonds, with an average of 371 ± 12.67, while the V433M variant exhibits fewer H-bonds, with an average of 350 ± 12.74. The lowest number of hydrogen bonds indicated an increase in the overall flexibility of CYP4F2 upon V433M variant.

**Fig. 4:**
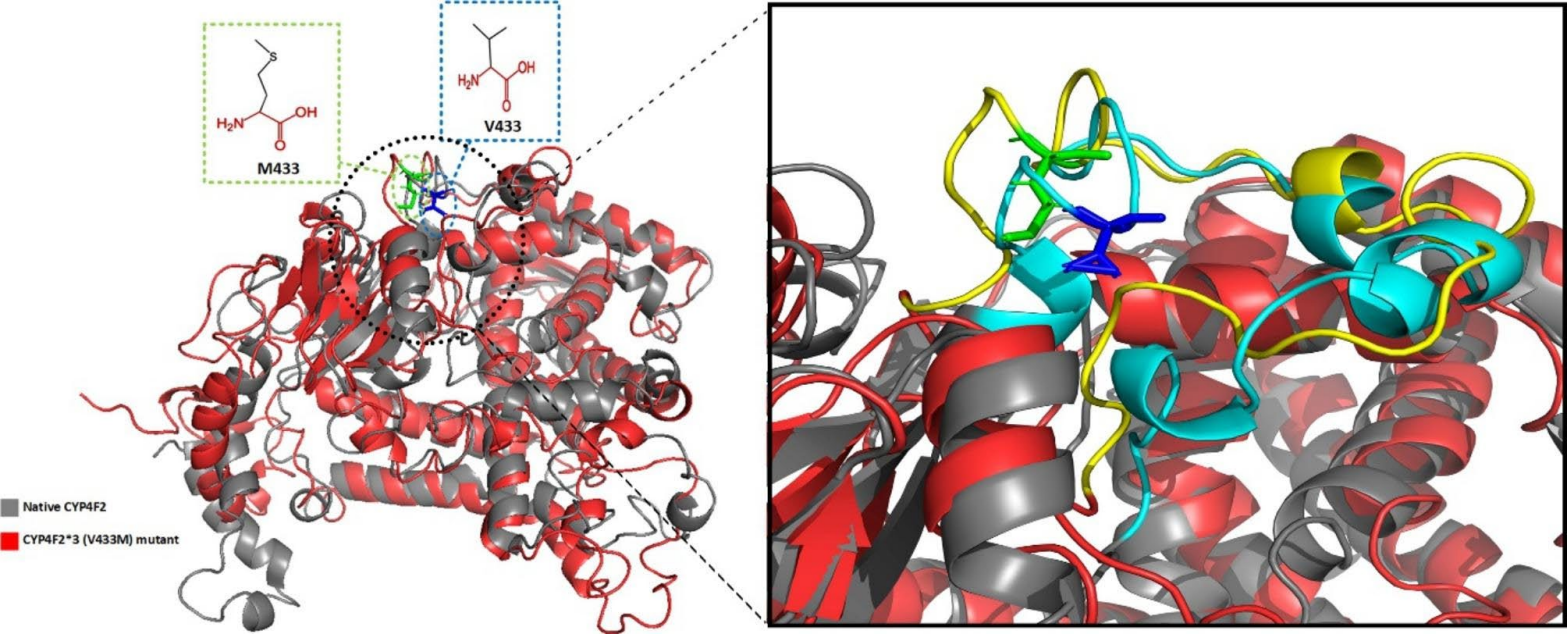
Superimposed structures of native CYP4F2 (black color) and CYP4F2*3 (V433M) variant (red color), the position of wild-type residue (V433) and mutant-type residue (M433) was characterized. The close-up view of V433 location illustrates that mutation to M433 caused changes from helix structure to turn which resulted in more flexibility in 445–460 region in mutant form compared to native. V433 shows in green stick and M433 shows in blue stick form. The 445–470 region is colored in yellow and cyan in mutant and native form, respectively

RMSF based on residue displacement explains the thermal stability, local flexibility, and heterogeneity of macromolecules over MD simulation time [58]. The RMSF plot indicated that residues in the N terminal region had a major difference in fluctuation between the native CYP4F2 structure and the V433M variant after 200 ns MD simulation. In addition, the V433M variant exhibited a higher fluctuation region in the residues 244–249 of the Gˊ helix and residues 251–260 of the G helix, followed by increased flexibility in the residues 400–470 when compared to the native CYP4F2 structure (Fig. 3E). It was also noticed that the average of RMSF was increased from 0.08 ± 0.03 in native CYP4F2 to 0.11 ± 0.07 in V433M variant, suggested that the V433M mutation altered structural flexibility. These data are compatible with the structural alignment of native and mutant forms after molecular dynamics simulation, indicating that mutant form had a more open structure than the native form especially in region 400–470 which near to site of mutation V433M and showed significant structure changes (Fig. 4).

**Fig. 5:**
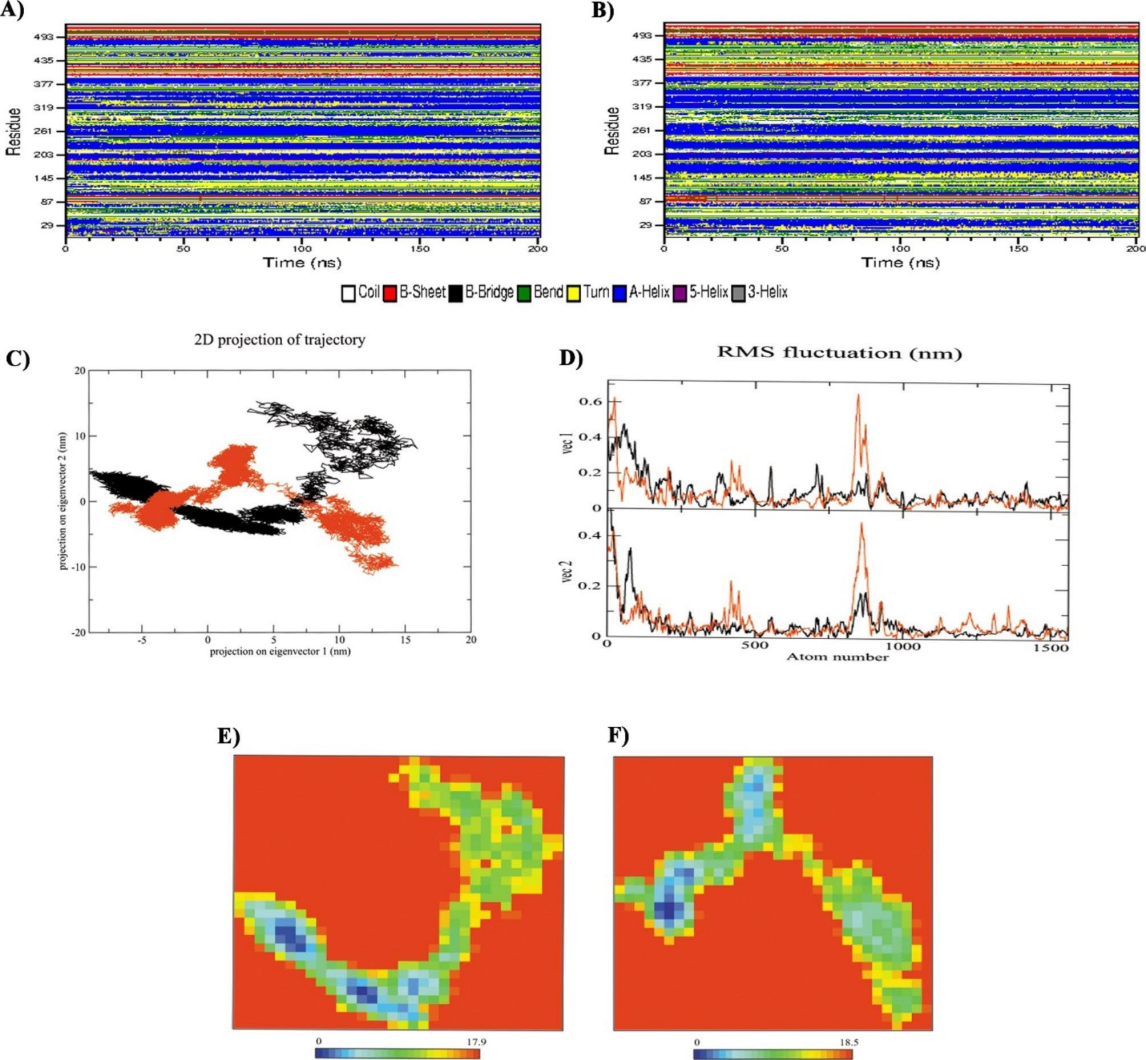
Secondary structure content in native CYP4F2 (**A**) and V433M variant (**B**) over 200 ns of the simulation period. PCA analysis plot of CYP4F2 in native (black) and V433M variant form (red) (**C**). The rmsf plot of eigenvector reveals V433M had higher fluctuation compared to native form (**D**). The Gibbs energy landscape plot during 200ns of simulation of native (E) and V433M variant form (**F**)

Changes in the secondary structure provide insight into the folding mechanism and conformational behavior of a protein. The DSSP plot (Fig. 5) showed that the native protein (Fig. 5A), and V433M variant (Fig. 5B) exhibited almost the same secondary structural arrangement. The data illustrated that native CYP4F2 contains an overall 60% secondary structure, including β-bridges, β-sheets, α-helices, and turns (Table 4). However, the V433M variant showed a slight decrease in the secondary structure profile compared that with of the native protein. Significant changes in the secondary structure content of the V433M variant can be seen due to bend and turn formation and loss of the A-Helix and 3-Helix without any change in β-sheets and coils.

**Table 4:** Secondary structure percentage of native CYP4F2 and V433M variant. * Structure = β-sheet + β-bridge + Turn + A-helix

Structure	Structure*	Coil	B-Sheet	B-Bridge	Bend	Turn	A-Helix	5-Helix	3-Helix
Native	60%	17%	8%	0%	16%	15%	37%	0%	7%
V433M	59%	17%	8%	0%	18%	16%	35%	0%	6%

PCA uses covariance matrices of Cα atoms to calculate the significant motions of atom pairs associated with vital biological functions. The first two principal components (PC1 and PC2) of the native and V433M variant proteins were generated by projecting the trajectories onto the respective eigenvectors. As seen in Fig. 5C, the V433M variant covered the slightly higher subspace spanned along the two eigenvectors (PC1: -3.56 nm to + 14.12 nm and PC2: -11.27 nm to + 8.76 nm) compared to the native (PC1: -2.31 nm to + 15.65 nm, and PC2: -5.2 to + 15.12 nm). It was also noticed that the trace of the diagonalized covariance matrix of the native form and V433M variant was calculated to be 64.13 and 69.99 nm, respectively. The rmsf eigenvector plot (Fig. 5D), based on atom number analysis, illustrated a higher V433M motion than the native form. In addition, the Gibbs free energy landscapes (FELs) of the first and second PCA showed that the V433M variant (Fig. 5E) had wider global energy minima (18.5 KJ/mol) than the native form (17.9 KJ/mol) (Fig. 5F), which indicates more conformational changes of the protein in the defined space. These results confirmed an increase in the overall flexibility of the V433M variant, which is in agreement with the RMSD, Rg, H-bonds, and RMSF values, as well as the SASA and DSSP analyses.

### Molecular docking

**Fig. 6:**
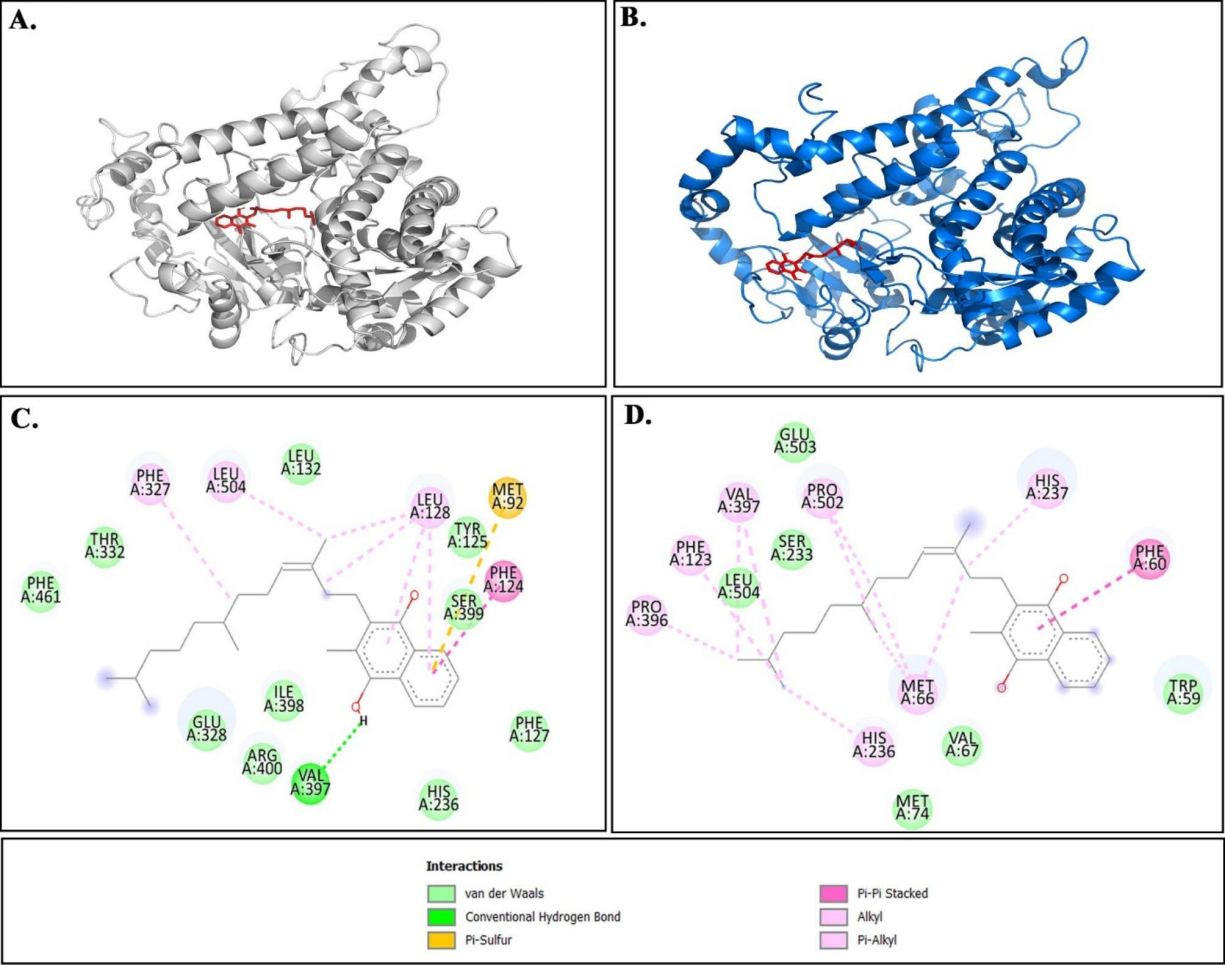
Interaction mode of VK1 with native CYP4F2 (**A**) and V433M variant form (**B**) in tertiary mode. Bond types analyses in native (**C**) and V433M variant form (**D**) using Discovery studio

The enzymatic activity of the CYP4F2 depends on its interaction with VK1. For this reason, molecular docking using the HADDOCK web server was performed to figure out the effect of the V433M variant on the interaction of the CYP4F2 protein with VK1 (Fig. 6). The binding site of VK1 on CYP4F2 protein was characterized according to a previous work of Li et al. (2018), that predicted binding site of VK1 in this protein. Based on this research, the amino acids including Trp59, Trp61, Met92 Phe124, His236, Phe327, Glu328, Val397, and Leu504 are responsible for interacting with VK1 [59]. The docking result (Table 5) showed that although the mutated form could still interact with VK1, it had a lower binding affinity (-8.5 ± 0.31 kcal/mol) than the native form (-10.0 ± 0.07 kcal/mol), which caused less activity of the mutated state compared to the native form. Bond-type analyses illustrated that in native form, VK1 formed strong forces such as hydrogen bonding, Pi-Pi stacking, and Pi-sulfur interactions with Val397, Met92 and Phe124 amino acids, whereas, in the mutant form, only Phe60 residue formed a Pi-Pi stacking with VK1. This caused VK1 to have more strong interaction with the native form than the mutant (Fig. 6).

**Table 5:** the docking result of CYP4F2 in both native and mutant form by HADDOCK webserver. Amino acids in bold are amino acids of CYP4F2 which are identified as important in interaction with VK1.

Complex structure	Amino acids in interaction with Vitamin K	∆∆G (Kcal/mol)
Native	Val397, **Phe327**, **Glu328**, Leu128, **Leu504**, Ser399, Tyr125, **His236**, Phe127, **Phe124**, Ile398	-10.00 ± 0.07
V433M	**His236**, **Val397**, Pro396, Ser323, Met66, **Leu504**, Pro502, Phe60, His327, Val67, **Trp59**	-8.5 ± 0.31

## Discussion

Several in vitro experiments have revealed that the CYP4F2*3 (V433M) variant is associated with decreased CYP4F2 activity and VK1 metabolism [14, 15, 21, 22]. McDonald et al.(2009) assessed that carriers of the CYP4F2*3 (V433M) variant allele have a reduced capacity for ω-hydroxylation VK1 [14]. Edson et al. (2013) also reported that the catabolism of menaquinone (vitamin K2) is reduced by the V433M mutation in CYP4F2 [15]. Furthermore, Stec DE et al. (2007) exploited the CYP4F2*3 (V433M) variant allele, which is linked to decreased biosynthesis of 20-hydroxyeicosatetraenoic acid (20-HETE) and increased hypertension risk [21]. A study by Zhang et al.(2017) suggested that the CYP4F2*3 (V433M) variant also affects mRNA expression levels of CYP4F2 [22].

The results of this computational study agree with previous experimental findings. According to these results, the CYP4F2*3 (V433M) variant had a significant damaging effect on the CYP4F2 protein structure.

In this study, five different stability prediction tools (I-mutant2, MUpro, DUET, SDM and mCSM) revealed a significant destabilizing structural effect of the CYP4F2*3 (V433M) variant on the CYP4F2 protein.

In the case of the V433M variant, the buried structural methionine residue was larger than the valine residue, which could disturb the interaction with other residues in the protein, as predicted by the Project HOPE. This finding is compatible with the structural analysis of the CYP4F2*3 (V433M) variant compared to the wild type using Chimera software, which showed that a larger side chain of methionine caused a clash score with neighboring residues, especially with the P104 residue. These data agree with the study of Cai et al. (2016) regarding the pathogenicity impact of the V139M variant on the PSEN2 protein, who discovered that both valine and methionine are nonpolar and hydrophobic residues, whereas methionine is larger in size. They suggested that the difference in the size of methionine and valine alters the interactions with neighboring amino acids and could result in extra stress inside the protein [60]. In addition, the results of the Project HOPE server determined that the V433 residue is positioned close to a highly conserved region and in contact with residues in a domain that is important for the activity of the protein. These data are in accordance with the ConSurf web server results, which illustrated that V433 is located near the conserved residues.

Based on the functional analysis of five different web servers, the CYP4F2*3 (V433M) variant was predicted to be a damaging amino acid variant compatible with the data of Naushad et al. (2021) using three tools (SIFT, PROVEAN, and PSEP), demonstrating the harmful impact of the CYP4F2*3 variant [61].

To further investigate the harmful effects of the CYP4F2*3 (V433M) variant, MD simulations and molecular docking analyses were performed. The apparent loss of stability and gain of flexibility were mainly observed in the RMSD and Rg plots, which were accompanied by a significant increase in SASA and a decrease in the number of hydrogen bonds for the V433M variant compared to native CYP4F2. We also found a relative increase in flexibility of residues 244–249 of the Gˊ helix, residues 251–260 of the G helix and residues 400–470 that was accompanied by the rise in bend and turn formation and decrease of A-Helix and 3-Helix conformation in the DSSP results of the V433M variant. PCA and FELs analyses also confirmed that the V433M variant showed a significant difference in overall flexibility compared to the native form, affecting protein folding, thereby decreasing the stability of the protein. As the proper structure of the protein is required for its activity, altering its conformational geometry could cause a loss of catalytic activity [62–64]. This result agrees with the molecular docking finding that the V433M variant showed lower binding affinity than the native form, which could influence the activity of the protein and impact the response to the warfarin dose.

## Conclusion

CYP4F2 is one of the critical CYP4 enzymes responsible for the metabolism of fatty acids, therapeutic drugs, and signaling molecules such as arachidonic acid, tocopherols, and vitamin K. Numerous studies have revealed that the missense variant CYP4F2*3 (V433M) causes decreased activity of CYP4F2 and inter-individual variations in warfarin dose in various ethnic groups. In the current study, we used various bioinformatics and computational tools to analyze the impact of the CYP4F2*3 (V433M) variant on the structure and function of the CYP4F2. Based on our findings, the CYP4F2*3 (V433M) variant is a damaging amino acid substitution with a destabilizing protein effect. Molecular dynamics simulation results showed that the V433M variant affected the dynamics and stability of CYP4F2 by decreasing the compactness and stability of the protein structure, which resulted in overall structural conformational changes and increased flexibility. The docking results showed that the CYP4F2*3 variant had a lower binding affinity between VK1 and CYP4F2, which caused lower activity of CYP4F2*3 compared to native CYP4F2. This study determined the molecular pathogenicity mechanism of the CYP4F2*3 variant on the human CYP4F2 protein and provided new information for understanding the structure-function relationship of CYP4F2 and other CYP4 enzymes. These findings will facilitate the development of effective drugs and individual treatment options.

## Data Availability

The datasets generated during and/or analyzed during the current study are available from the corresponding author on reasonable request.

## List of abbreviations

CYP2C9: Cytochrome P450 2C9
CYP4F2: Cytochrome P450 4F2
FEL: Free energy landscapes
fs: Femtoseconds
MD: Molecular dynamic
NPT: Constant number of particles, pressure, and temperature
NVT: Constant number of particles, volume, and temperature
nm: Nanometer
ns: Nanoseconds
OPLS: Optimized potential for liquid simulations
PDB: Protein Data Bank
PCA: Principal component analysis
RMSD: Root means square deviation
RMSF: Root mean square fluctuation
Rg: Radius of gyration
SASA: Solvent accessible surface area
SPC: Simple point charge
VIF: Variance inflation factor
VKORC1: Vitamin K epoxide reductase complex 1
